# Communicating study results to our patients: Which way is best?

**DOI:** 10.4103/0019-5413.40249

**Published:** 2008

**Authors:** BA Petrisor, Paul Tornetta III

**Affiliations:** McMaster University, Canada; * Boston University, USA

**Keywords:** Communication, evidence-based medicine, number needed to treat, odds ration, relative risk

## Abstract

Before we are able to communicate evidence and evidence results to patients we must first be familiar with the common ways by which results may be presented to our patients. We describe five approaches (relative risk, risk reduction, odds ratio, absolute risk difference and number needed to treat) of transforming the results of an orthopaedic study for communication with patients.

## INTRODUCTION

Evidence based medicine (EBM) is described as the “conscientious, explicit and judicious use of current best evidence in making decisions about the care of individual patients”.^1^ The implications of this statement suggest that physicians must be able to not only identify, appraise and subsequently understand the literature around a treatment topic, but integrate this literature into their own clinical algorithms and judgement and subsequently apply this knowledge in the care of individual patients. An important tenet of EBM however is not only taking into account literature based treatment recommendations in the care of patients but (and potentially most importantly) taking into account the values and preferences of the individual we are treating. While this sounds relatively straightforward there are many ways this integration of knowledge and literature and subsequent action in the form of treatment recommendations can break down. As part of understanding and critically appraising the literature around a topic, we as surgeons need to understand how the results of studies have been expressed and interpreted. This helps us to ascertain if the results are applicable to our patient population and more specifically to the individual patients we are treating. Once this is done, these results need to be conveyed to our patients who come from all backgrounds have different values, priorities, preferences and while some have medical knowledge arguably most do not. Thus in order to fully understand patients’ values and preferences we must be able to discuss fully the risks and benefits of a proposed treatment with them, as well as the treatment alternatives. These risks and benefits are derived from the results of studies on patients similar to the patient(s) we may be treating. This paper will focus on how we as surgeons can communicate with patients to increase their understanding of a problem and include them in the decision making process.

## PRESENTING RESULTS

Before we are able to communicate evidence and evidence results to patients we must first be familiar with the common ways by which results may be presented. For the purposes of discussion we will focus on “dichotomous” data, that is, either an event happens or it does not happen and use an example from the orthopaedic literature

One can see by this example that there are multiple ways that dichotomous data can be presented with most of these ways being probabilities. For example, the risk of developing a nonunion with operative management of a clavicle fracture is reduced by 79% [Relative Risk Reduction (RRR)]. Said another way, one is 79% less likely to develop a nonunion with operative management. Probabilities however can lead to some confusion in their interpretation.^2^,^3^ Indeed Hoffrage and Gigerenzer found that only 1 out of 24 physicians were able to give a correct answer when statistical information for a clinical problem was presented as probabilities. With the same clinician population, when the probabilities were presented as natural frequencies, 16 out of 24 physicians were able to give the correct answer.^3^ Secondly, terms such as relative risk or relative risk reduction may erroneously elevate the perceived impact of a treatment effect.^4^,^5^ A 79% reduction in the risk of developing a nonunion with operative management sounds like a treatment effect and could potentially really argue in favour of an operation. However, if a patient's inherent or baseline risk of developing a nonunion without treatment is only 2% then reducing this risk by 79% takes the patient's risk of nonunion to just below 1%, which is not a very clinically significant reduction in risk. This makes a potentially significant sounding treatment effect potentially not very clinically significant in either a surgeon's or an individual patient's mind. This then illustrates one potential problem in communicating results; if clinicians have difficulty understanding probabilities, how then are we to explain these probabilities to patients?

Representing data as a number needed to treat makes a more clinically relevant and potentially powerful statement.^5^ To say, for every 9 patients treated with an operation one nonunion can be prevented allows for an immediate interpretation of the clinical impact of a treatment.^5^ One caveat in using the number needed to treat is that adjustments need to still be made according to the patient's baseline risk.^5^

Given that there are multiple ways that data can be presented to the medical community, we will now explore how this can be translated and communicated effectively to our patient population which is varied and in general have a lack of formal medical knowledge.

## COMMUNICATING RESULTS TO PATIENTS

As surgeons, we must necessarily develop a relationship with patients. This relationship can take many forms from a purely paternalistic one (in which the clinician makes the treatment decisions) to a completely patient independent model (in which the clinician delivers the facts to the patient and they then make the decision regarding treatment).^6^,^7^ It seems that the most conducive relationship for communicating with patients lies somewhere in between.^8^ Epstein *et al.*, suggest that a relationship centered model “provides opportunities for information transfer but also enhances the patient's ability to participate in care”.^8^ This is done by the active encouragement of patients' participation in the decision making process. They argue that it is important to not only involve patients and their families in the decision making process but also to not exclude the importance of a clinician's judgment (such as with the informed choice or patient independent model) and experience as it relates to the condition and the medical literature.^8^ Indeed, when in the context of discussing risk with patients, it has been suggested that communication should be a “two way process”.^9^ This is best facilitated with a relationship centered model of care.^9^

### Tools for communicating results to patients

Are there tools that may be effective for clinicians to use when discussing the evidence with patients? Table 1 lists the tools that have been examined in the literature. Trevena *et al.*, have conducted a systematic review of studies looking at the effectiveness of these tools.^11^ They suggest that the very act of communicating with patients about evidence increases patient understanding.^11^ While this sounds intuitive, one study suggests that physicians ask patients if they have questions regarding a treatment in less than half of outpatient visits.^12^ While Broddock *et al.*, found that discussions around patient understanding of risks and benefits of a treatment were rare.^13^ This is compounded by the fact that patients may not always “hear” what is being presented to them in a clinician patient encounter.^14^ If we think of a busy orthopaedic fracture clinic how much time is actually allotted to each patient?

**Table 1:** Nonoperative treatment compared with plate fixation of displaced midshaft clavicular fractures. A multicenter, randomized clinical trial.^2^

	Operative treatment	Nonoperative treatment
Number of patients	62	49
Number of nonunions	2	7
Event rate for nonunion	2/62 = ∼3%	7/49 = ∼14%
Natural frequency event rate	3 people out of 100 will develop a nonunion with operative management	14 people out of 100 will develop a nonunion with nonoperative management
Relative risk (RR) of nonunion in operative group as compared to nonoperative group	3%/14% = 0.03/0.14 = 0.21	
Relative risk reduction (RRR) of developing a nonunion with operative management	1 - 0.21 = 0.79 = 79%	
Odds of developing a nonunion in the operative group as compared to the nonoperative group (OR)	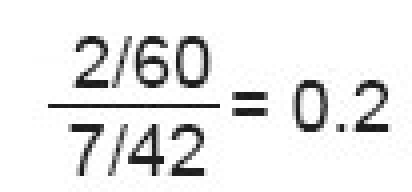	
Absolute risk reduction (ARR)	0.03 − 0.14 = 0.11 = 11%	
Number needed to treat (NNT) (for every patient treated with operative fixation one nonunion is prevented)	1/ARR = 1/0.11 = 9	

From the list provided in table 1, Trevena *et al.*, suggest that either verbal, written or video information presented in a structured format and specifically tailored to the individual patient provides for a better patient understanding of the evidence.^11^ This concept of personalized information has also been addressed by Edwards *et al.*, in a recent Cochrane Collaboration systematic eview.^15^ They suggest that from the available literature on patient's informed decision making as regards taking screening tests that personalized risk communication (that is taking into account a patient's baseline risk of disease etc.) increases patient's willingness to undergo screening tests (odds ratio 1.13, 95% CI 1.02 to 1.24).^15^ This harks back to our own understanding of the interpretation of results. That is to say that the results obtained from the literature reflect the sample pool of patients used in the particular studies. This sample of patients may have a different risk profile than an individual patient we are treating. Thus we need to understand our individual patient's baseline characteristics for our own understanding of the applicability of results. More importantly however, it may be that we need to include these characteristics in our patient tailored discussion of the risks and benefits of a proposed treatment.

### Communicating probabilities

Given the fact that results are presented in many cases as probabilities [Relative Risk (RR), RRR or Absolute Risk Reduction (ARR)] and tools in the form of patient oriented communication are effective aids, are there ways of presenting probabilities to patients that may increase their inherent understanding? In their systematic review of the literature Trevena *et al.*, suggest that natural frequency event rates may be a more effective way of communicating probabilites.^11^ Similarly, Gigerenzer argues that probabilities such as relative risk are potentially prone to misinterpretation as they refer to specific but unknown reference classes.^5^ As an illustration, from our example the relative risk of 0.21 or the relative risk reduction of 79% is based on the sample of people in that study. That is, the study sample is the reference class, however when one says “your risk of nonunion will be reduced by 79% if you have an operation” to a patient, the reference class is unknown and we then need to relate it to the patient's individual risks and characteristics. This is why the number needed to treat is powerful as it is derived from the absolute risk; therefore the reference class is known. That is, for every 9 people with a clavicle fracture treated with an operation, one nonunion will be prevented. Does this mean that the NNT is the best way to discuss treatment effects with patients however? Possibly not as Christensen *et al.*, suggest that the NNT may not easily be interpreted by non-medical patients.^16^ They put forward that using the postponement of harm to measure the effectiveness of treatment may be more beneficial to patients.^16^ Similarly Sheridan *et al.*, have found that patients showed less of an understanding of the NNT as compared to the RR. However, importantly this was only when baseline characteristics and risk were presented with the RR. These studies suggest that while it may be more clinically relevant for surgeons to use NNT when interpreting study results, this may not translate when communicating with patients.

Another strategy in communicating probabilities to patients is to use illustrations and graphs.^17^ Using bar charts or pictographs may enhance patients understanding of risk over other types of graphical data presentation.^10^,^17^ A pictograph is a graph that can pictorially represent natural frequency event rates. For example a box of 100 squares with 3 shaded would represent pictorially 3 people out of 100 having a specified event (from our previous example 3 people out of 100 would develop a nonunion with operative management). Price *et al.*, suggest that horizontal pictographs presenting natural frequency event rates were perceived more accurately than vertical ones.^17^

### Communicating probabilities to orthopaedic patients: Do the same rules apply?

Bhandari and Tornetta reported a face-to-face survey administered to 50 patients attending the fracture clinic at a university-affiliated hospital.^18^ Patients were asked to consider a scenario (hip fracture) and to decide which treatment alternative they preferred based upon risk presentation. The authors developed this single scenario aimed at identifying how patients’ perceptions about having alternative surgical procedures changed by the manner in which data was presented. The questionnaire was piloted among three surgeons and five patients to ensure clarity. Risk was presented in five ways: (1) absolute risk difference (i.e, 9–6% = 3%), (2) relative risk reduction [(1–6%/9%) _100 = 33%], (3) relative risk [6%/9% = 0.66], (4) number needed to treat (NNT) [1/0.03 = 33] and (5) odds ratio [AD/BC = 0.67]. Patients were most likely to favor internal fixation when the mortality results comparing internal fixation versus arthroplasty were presented as a relative risk reduction (internal fixation may reduce the risk of mortality by 33% when compared with arthroplasty). Patients continued to favor internal fixation despite being presented with a significantly increased risk of revision surgery? Internal fixation definitely increases the risk of revision surgery by > 100% (proportion of patients still favoring internal fixation = 62%, 95% confidence interval 48?80%). These findings confirm that orthopaedic patients are subject to the same influences of risk communication. Surgeons must use care in utilizing relative risk reductions in the absence of actual risk data given the presented findings that may overestimate the relative benefits of one procedure over another.

### Patient Communication Strategies

Epstein *et al.*, have put forward an algorithm for discussing evidence with patients.^9^ This can be used to set the stage for the entire patient encounter with the discussion of evidence as but one component of the interaction. This is but one potential method and one would argue that clinicians require flexibility when incorporating this strategy into their practices. Indeed the oft quoted adage “everyone is different” plays a role here and it behooves us as clinicians to have a number of tools at the ready.

## CONCLUSION

With the increased use of the principles of EBM in clinical decision making, it is important for clinicians to remember that within the framework of EBM, understanding patients’ values and preferences has a significant role. Surgeons must find strategies to effectively communicate evidence to patients such that they can be actively involved in the decision making process. It is suggested that taking the time for the discussion and potentially using aids to express evidence as natural frequencies may go a long way to help our patients reach decisions regarding a proposed treatment. However it is also important that this be done within the context of a solid physician-patient relationship that remains open to dialogue. It is founded on trust and instills confidence in patients with the treatment decisions that have been made.
